# Long non-coding RNA PVT1 regulates the migration of hepatocellular carcinoma HepG2 cells via miR-3619-5p/MKL1 axis

**DOI:** 10.17305/bjbms.2020.4641

**Published:** 2021-04

**Authors:** Hua Liu, Yan Yin, Ting Liu, Yanying Gao, Qing Ye, Junqing Yan, Fushuang Ha

**Affiliations:** 1 The Third Central Hospital of Tianjin, Tianjin, China; 2 Tianjin Key Laboratory of Extracorporeal Life Support for Critical Diseases, Tianjin, China; 3 Artificial Cell Engineering Technology Research Center, Tianjin, China; 4 Tianjin Institute of Hepatobiliary Disease, Tianjin, China; 5 Respiratory and Critical Care Medicine of Tianjin Chest Hospital, Tianjin, China; 6 Tianjin Institute of Cardiovascular Disease, Tianjin Chest Hospital, Tianjin, China

**Keywords:** PVT1, miR-3619-5p, MKL1, cell migration, hepatocellular carcinoma, HCC

## Abstract

Hepatocellular carcinoma (HCC) is the third most common malignant tumor of the digestive system. Plasma cell tumor heterotopic gene 1 (PVT1) is an intergenic long non-coding RNA that is aberrantly expressed in different cancers. Myocardin-related transcription factor A or megakaryoblastic leukemia 1 (MKL1) is a transcriptional coactivator of serum response factor that has been shown to promote cancer cell migration and invasion. In this study, we investigated the relationship between PVT1 and MKL1 as a novel regulatory mechanism underlying HCC progression. We used HepG2 and Cos-7 cell lines. Transfection experiments with miR-3619-5p mimics/inhibitor, PVT1, siRNA-PVT1, MKL1, or siRNA-MKL1 were performed. RNA and protein levels were analyzed by quantitative reverse transcription PCR and Western blot, respectively. Cell migration was assessed by transwell assay. Luciferase assays, RNA-FISH, RNA immunoprecipitation, and chromatin immunoprecipitation assays were performed to confirm the interaction between PVT1, miR-3619-5p, and MKL1 in HCC cells. Overexpression of PVT1 was positively correlated with MKL1 upregulation, which promoted HepG2 cell migration. miR-3619-5p inhibited MKL1 expression in HCC cells by acting on its 3′-UTR. Furthermore, PVT1 promoted MKL1 expression and migration in HCC cells by directly binding to miR-3619-5p. In a positive feedback loop, MKL1 could activate PVT1 transcription by binding to the CArG box in the promoter region. Our findings may provide a basis for the development of novel targeted therapies in HCC.

## INTRODUCTION

Hepatocellular carcinoma (HCC) gradually became one of the most common cancers and the second leading risk around the world [1]. Most HCC patients eventually die due to tumor metastasis-associated deaths [2]. Today, screening novel biomarkers for developing effective therapeutic targets against the metastasis of HCC may make up for the shortcomings of current therapeutic strategies.

Human plasma cell tumor heterotopic gene 1 (PVT1) belongs to intergenic gene long non-coding RNAs (lncRNAs). In mice, this gene site frequently produces multiple translocations [3]. In addition, the same phenomenon can be detected in one region of the human PVT1 gene in Burkitt’s lymphoma [4]. PVT1, located at 8q24.21, has a complexity in its structure that is revealed gradually, with the development of high flux technology [5]. The existence of an aberrant expression of PVT1 in many human cancers has been reported recently. PVT1 may act as a noninvasive diagnostic biomarker in small cell lung cancer because its overexpression always means a poor prognostic [6]. Moreover, PVT1 can regulate forkhead box M1 (FOXM1) to facilitate gastric cancer growth and invasion [7]. As a lncRNA, PVT1 can also affect miRNAs directly, acting as a molecular sponge to regulate cancer cell development similar to miR-195 in osteosarcoma [8].

Megakaryoblastic leukemia 1 (MKL1) is a transcriptional coactivator of serum response factor (SRF), which can bind to the CArG box (CC(A/T)_6_GG) in the promoters of various immediate-early and muscle-specific genes [9-11]. In a previous report, MKL1 could also promote cancer cell migration and invasion [12], which has a similar function as PVT1. However, the relationship between the two factors is still unknown.

Therefore, the aim of this study is to investigate the relationship between PVT1 and MKL1 to reveal new molecular regulatory mechanisms in metastasis of HCC.

## MATERIALS AND METHODS

### Cell culture

The human HCC cell line HepG2 and Cos-7 cells used in this study were purchased from ATCC (Manassas, VA, USA; nos. HB-8065 and CRL-1651, respectively). These cells were seeded in Dulbecco’s Modified Eagle Medium (DMEM, Gibco; Thermo Fisher Scientific Inc., Waltham, MA, USA) supplemented with 10% fetal bovine serum (FBS, Gibco; Thermo Fisher Scientific Inc., Waltham, MA, USA) at 37°C in humidified air with 5% CO_2_.

### Cell transfection

For the transfection experiments, the HepG2 cells were cultured in a medium without FBS for 0.5 h and then transfected with a transfection reagent (FuGENE^®^ HD; Roche Diagnostics, Basel, Switzerland). After incubation for 6 h, the medium was replaced with fresh complete medium for 48 h. MiR-3619-5p mimics/inhibitor, siRNA-PVT1 (si-PVT1), siRNA-MKL1 (si-MKL1-1, si-MKL1-2, and si-MKL1-3), and MKL1 were purchased from Guangzhou RiboBio Co. Ltd. (Guangzhou, China) and GeneChem Co. Ltd. (Shanghai, China).

### Quantitative reverse transcription polymerase chain reaction (qRT-PCR)

The total RNA was isolated using TRIzol reagent (Invitrogen, Waltham, MA, USA) and reverse-transcribed using M-MLV reverse transcriptase (Promega Corporation, Madison, WI, USA). Quantitative PCR was carried out using the Fast SYBR^®^ Green Master Mix (Applied Biosystems; Thermo Fisher Scientific Inc., Waltham, MA, USA). Glyceraldehyde 3-phosphate dehydrogenase (GAPDH) was used as control to show equal loading of the mRNA. The data were shown as relative expression levels after being normalized by GAPDH. For the detection of miR-3619-5p by qRT-PCR, miRNA was extracted using the miRNA Kit (Omega Bio-Tek Inc., Norcross, GA, USA), according to the manufacturer’s instructions. Then, miRNA was reverse-transcribed into first-strand cDNA with miRNA 1^st^ Strand cDNA Synthesis Kit (by stem-loop) (Vazyme; Nanjing, China). Quantitative PCR was then carried out using the Fast SYBR^®^ Green Master Mix (Applied Biosystems; Thermo Fisher Scientific Inc., Waltham, MA, USA). The U6 gene was used as the endogenous control gene for normalizing the expression of miR-3619-5p. The primers for the PCR analysis are listed in Table 1. The thermocycling conditions were as follows: 95°C for 5 min followed by 40 cycles at 95°C for 10 sec and 60°C for 30 sec, then a melting curve analysis from 60°C to 95°C every 0.2°C for 1.5 min was obtained. Each sample was analyzed in triplicate and quantified using the 2-^DDCq^ method [13].

**TABLE 1:**
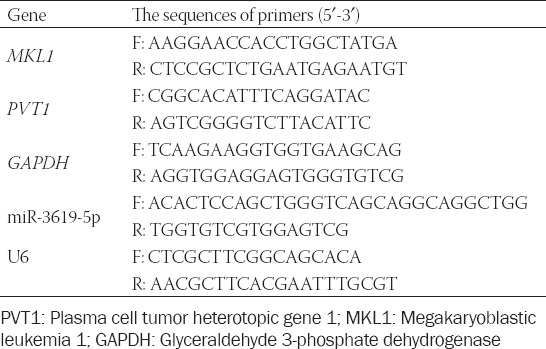
Sequences of primers used in quantitative reverse transcription polymerase chain reaction

### Protein extraction and Western blotting

The proteins were separated by sodium dodecyl sulphate-polyacrylamide gel electrophoresis (SDS-PAGE) and transferred to nitrocellulose membranes. The membranes were immunoblotted with anti-MKL1 antibody (dilution 1:1,000; cat. no. 14760; Cell Signaling Technology Inc., Danvers, MA, USA) or anti-GAPDH antibody (dilution 1:2,000; cat. no. 97166; Cell Signaling Technology Inc., Danvers, MA, USA) overnight at 4°C and then incubated with IRDye™-800 conjugated anti-mouse secondary antibody (dilution 1:5,000; cat. nos. 115-005-146; Jackson ImmunoResearch Laboratories Inc., West Grove, PA, USA) for 60 min at room temperature. The specific proteins were visualized using Odyssey Infrared Imaging System (LI-COR Biosciences, Lincoln, NE, USA). GAPDH expression was used as control to show the equal loading of the protein samples.

### Transwell assay

The invasion assay was performed using transwell chambers (Corning Incorporated, Corning, NY, USA) that had Matrigel (50 μL; BD Biosciences, New Jersey, USA) pre-coated polycarbonate membranes (8.0 μm pore size). A total of 1 × 10^4^ cells were added to the upper chamber with FBS-free culture medium. The lower chamber was filled with 500 μL complete medium. Following incubation for 24 h, the cells on the lower surface of the membrane were fixed in 4% paraformaldehyde and stained with 0.1% crystal violet. The cells were counted in five random microscopic fields (magnification, ×20). Finally, the crystal violet was washed with 33% acetic acid and measured at 570 nm.

### Luciferase constructs, site mutation, and luciferase assay

The fragment containing PVT1, pmirGLO-PVT1-WT, was fused to a pmirGLO vector. pmirGLO-PVT1-Mut is similar to pmirGLO- PVT1-WT, with the mutated binding site of miR-3619-5p. Cos-7 cells (2 × 10^5^/well) were cotransfected with miR-3619-5p mimics or its respective negative control RNA (Guangzhou RiboBio Co. Ltd., Guangzhou, China) in combination with pmirGLO-PVT1-WT or pmirGLO-PVT1-Mut. After 48 h, the Dual Luciferase Assay System (Promega Corporation, Madison, WI, USA) was used to test luciferase activity. PGL3-PVT1-WT: PVT1 5′-flanking region (−1000/0) was fused to pGL-3 luciferase coding sequence. PGL3-PVTI-Mut is equal to pGL-3-PVT1-WT, except that the MKL1-binding CArG box site is mutated from CCTATTTTGC to TTTATTTTAA. Cos-7 cells (2 × 10^5^/well) were cotransfected with MKL1 or its control plasmid (pcDNA3.1-) in combination with pGL3-PVT1-WT or pGL3-PVT1-Mut. After 48 h, the Dual Luciferase Assay System was used to test luciferase activity. The results were expressed as a fold induction relative to the cells transfected with the control after normalization to Renilla activity. For dual luciferase assay results, all columns represent the mean result of three independent experiments, and the error bars represent standard deviation.

### RNA fluorescence in situ hybridization (RNA-FISH)

A Cy3-labeled lncRNA-PVT1 complementary DNA probe was synthesized and used for RNA-FISH (GeneChem Co. Ltd., Shanghai, China). The procedures were performed as previously described with slight modification [14].

### RNA immunoprecipitation (RIP)

RNA immunoprecipitation experiments were performed as previously described [15]. For anti-protein argonaute-2 (anti-Ago2) RNA immunoprecipitation (RIP), 2 × 10^7^ HepG2 cells were used with the RIP kit (Cell Signaling Technology Inc., Danvers, MA, USA). Normal rabbit immunoglobulin G (IgG) antibody was used as negative control. The final results were detected by qRT-PCR. The gene-specific primer used for detecting lncRNA PVT1 is provided in Table 1.

### MS2-RIP

MS2 (Addgene, Watertown, Massachusetts, MA, USA), MS2-PVT1, and MS2-PVT1-Mut were used for this test. The three plasmids were transfected into HepG2 cells. After 48 h of transfection, the HepG2 cells were used to perform RIP experiments as previously described [16]. The RNA fraction isolated by RIP was analyzed using qRT-PCR. The gene-specific primer used for detecting miR-3619-5p is provided in Table 1.

### MiR-3619-5p mimics and miR-3619-5p inhibitor

MiR-3619-5p mimics, miR-3619-5p inhibitor, and the negative control (control or NC) were purchased from RiboBio. The cells in the six-well plates were transfected with the mimics at 100 nM. The transfection was performed using riboFECTTM CP Reagent (Guangzhou RiboBio Co. Ltd., Guangzhou, China) according to the manufacturer’s instructions.

### Chromatin immunoprecipitation (ChIP) assay

The researchers used a commercial ChIP assay kit (Merck KGaA, Darmstadt, Germany) following the manufacturer’s instructions. Each test group was incubated with 1% formaldehyde to cross-link DNA-protein complexes. After washing with ice-cold phosphate buffer saline (PBS) for 3 times, the cells were lysed in SDS lysis buffer. The lysate was then sonicated to shear DNA to 200–1000 bp fragments. Then, anti-MKL1 antibody (cat. no. 14760; Cell Signaling Technology Inc., Danvers, MA, USA) was used to immunoprecipitate the cross-linked protein at 4°C overnight. IgG acted as negative control. The DNA was used as a template for qRT-PCR and utilized the MKL1 binding sites. The PCR primer sequences were forward, 5′- AAATGTGTTTGTGTGTGGTGG-3′ and reverse, 5′-TCAACTTGTCGCTG CCAGTAT-3′.

### Statistical analysis

Data were expressed as mean ± SEM, accompanied by the number of experiments performed independently. Statistical analysis of the differences between the two groups was performed using Student’s *t*-test. A one-way analysis of variance followed by Tukey test was performed to compare differences among multiple groups. The differences at *p* < 0.05 were considered statistically significant.

## RESULTS

### PVT1 can promote migration of HepG2 cells via MKL1

**FIGURE 1:**
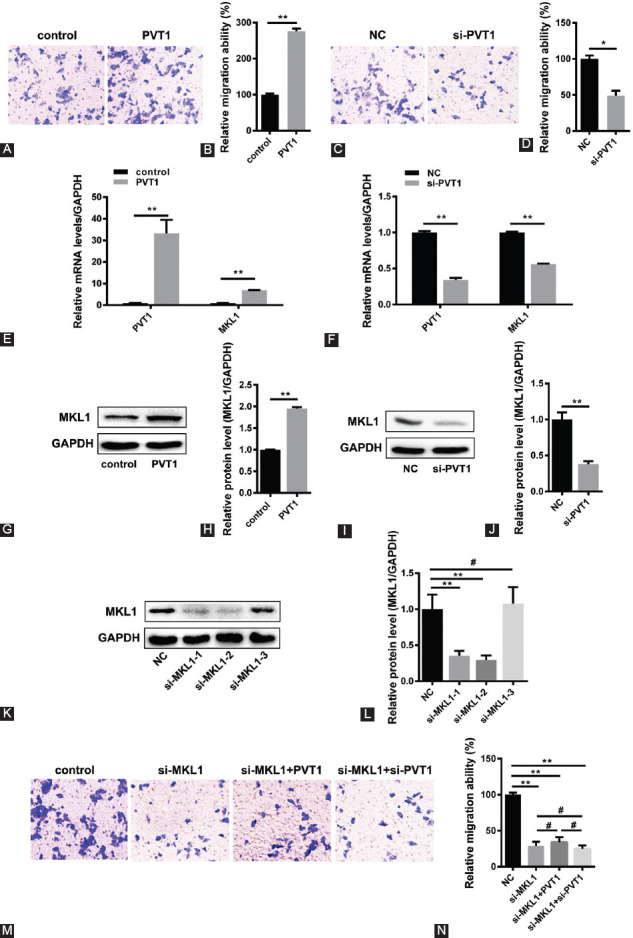
Plasma cell tumor heterotopic gene 1 (PVT1) can promote the migration of HepG2 cells via megakaryoblastic leukemia 1 (MKL1). (A, B, C, and D) The effect of PVT1 overexpression or silencing on HepG2 cell migration was measured by transwell assay (*n* = 3, *p < 0.05, **p < 0.01). (E and F) Quantitative reverse transcription polymerase chain reaction was performed to quantitatively measure MKL1 mRNA levels after overexpression or knockdown of PVT1 in HepG2 cells (*n* = 3, **p < 0.01). (G, H, I, and J) Following the overexpression or knockdown of PVT1 in HepG2 cells, the protein levels of MKL1 were examined through Western blot. A representative image of the Western blot of MKL1 protein expression (G and I) and the quantitation (H and J) (*n* = 3, **p < 0.01). (K and L) Western blot was used to detect the effect of MKL1 inhibitor in HepG2 cells. A representative image of the Western blot of MKL1 protein expression (K) and the quantitation (L) (*n* = 3, **p < 0.01, ^#^*p* > 0.05). The researchers chose si-MKL1-2 for follow-up experiments to knockdown endogenous MKL1. (M and N) In the absence of an endogenous MKL1, transwell assay was used to detect HepG2 cells migration ability with PVT overexpression or silencing (*n* = 3, **p < 0.01, ^#^*p* > 0.05).

To illustrate the possible function of PVT1 in HepG2 cells, the researchers facilitated PVT1 overexpression or silencing in HepG2 cells. The transwell results showed that PVT1 can inhibit the migration of HepG2 cells (Figure 1A-D). Moreover, qRT-PCR and Western blot were used to reveal the potential relationship between PVT1 and MKL1. The data showed that the expression of MKL1 was upregulated when PVT1 was overexpressed. Similarly, when PVT1 was silenced, the expression of MKL1 was depressed, suggesting that PVT1 promoted the migration of HepG2 cells, which may be due to the fact that MKL1 expression had a positive correlation with the expression of PVT1 (Figure 1E-J). Further experiments confirmed our results. When MKL1 was knocked down, PVT1 lost its ability to regulate the migration of HepG2 cells (Figure 1K-N). These results indicate that PVT1 can promote the migration of HepG2 cells via MKL1.

### MKL1 can be regulated by miR-3619-5p

**FIGURE 2:**
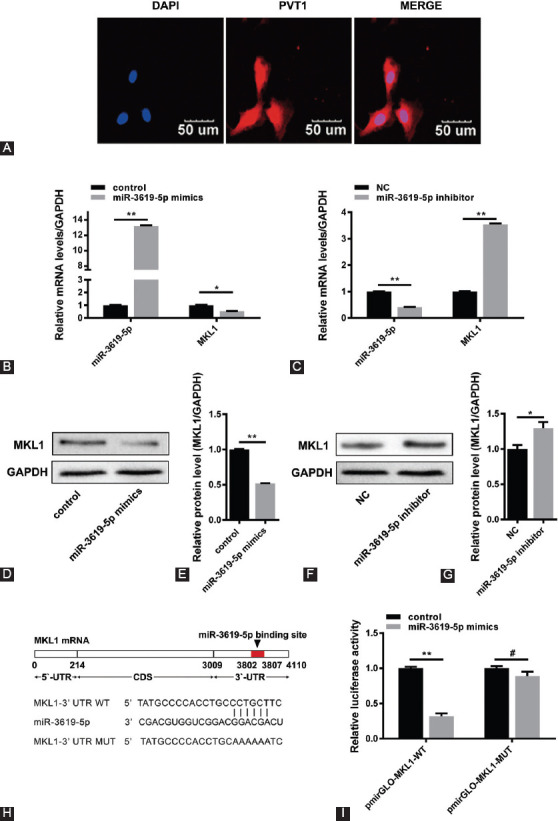
Megakaryoblastic leukemia 1 (MKL1) can be regulated by miR-3619-5p. (A) Localization of plasma cell tumor heterotopic gene 1 (PVT1) by RNA fluorescence in situ hybridization in HepG2 cells. The nuclei were stained blue (DAPI). Scale bars represent 50 mm. (B and C) The mRNA levels of MKL1 were measured by quantitative reverse transcription polymerase chain reaction in HepG2 cells which were transfected with miR-3619-5p mimics or inhibitor (*n* = 3, *p < 0.05, **p < 0.01). (D, E, F, and G) Following overexpression or knockdown of miR-3619-5p in HepG2 cells, the protein levels of MKL1 were examined through Western blot. Representative image of the Western blot of MKL1 protein expression (D and F) and the quantitation (E and G) (*n* = 3, *p < 0.05, **p < 0.01). (H) Schematic representation of the predicted binding sites for miR-3619-5p in MKL1 3′-untranslated region (UTR). (I) Luciferase assay was used to prove the binding sites between miR-3619-5p and MKL1 3′-UTR in Cos-7 cells (*n* = 3, **p < 0.01, ^#^*p* > 0.05).

The FISH experiment proved that PVT1 can be localized in the cytoplasm, suggesting that PVT may act as a competing endogenous RNA (ceRNA) on some miRNAs regulating the expression of MKL1 (Figure 2A). Based on the results, and following the bioinformatics analysis (using online TargetScan Software, available at http://www.targetscan.org), we found that miR-3619-5p may be a potential target. Then, qRT-PCR and Western blot results showed that miR-3619-5p can inhibit the expression of MKL1 (Figure 2B-G), and the molecular mechanism of this process was that miR-3619-5p can degrade the MKL1 3′-untranslated region [UTR] (Figure 2H-I).

### PVT1 can promote MKL1 expression and migration of HepG2 cells through miR-3619-5p

**FIGURE 3:**
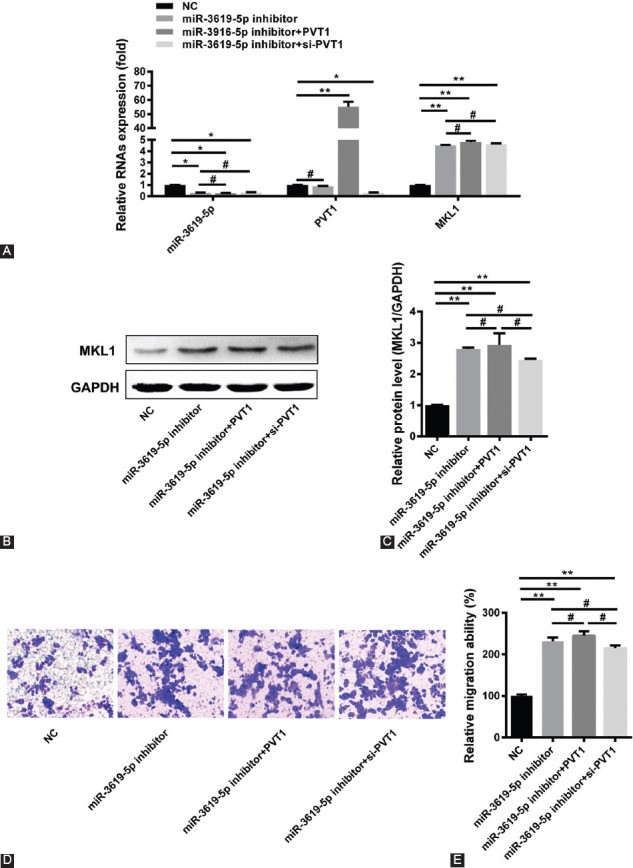
Plasma cell tumor heterotopic gene 1 (PVT1) can promote megakaryoblastic leukemia 1 (MKL1) expression and migration of HepG2 cells through miR-3619-5p. (A) Quantitative reverse transcription polymerase chain reaction was used to detect whether PVT1 can regulate MKL1 expression after knocking down miR-3619-5p in HepG2 cells (*n* = 3, *p < 0.05, **p < 0.01, ^#^*p* > 0.05). (B and C) Using miR-3619-5p inhibitor to silence endogenous miR-3619-5p in HepG2 cells, the expression of MKL1 was measured by Western blot with PVT1 overexpression or silencing. A representative image of the Western blot of MKL1 protein expression (B) and quantitation (C) (*n* = 3, **p < 0.01, ^#^*p* > 0.05). (D and E) After knocking down miR-3619-5p, the effect of PVT1 on HepG2 cells migration was detected by transwell assay (*n* = 3, **p < 0.01, ^#^*p* > 0.05).

To explore whether miR-3619-5p is the target of PVT1, qRT-PCR and Western blot testing were used to explore the expression of MKL1 in HepG2 cells with knocked-down miR-3619-5p. The results showed the overexpression or silencing of PVT1 in HepG2 cells with miR-3619-5p knocked down cannot regulate MKL1 expression (Figure 3A-C). Moreover, the transwell test showed that PVT1 lost the ability to affect HepG2 cell migration after miR-3619-5p was knocked down (Figure 3D,E). Evidently, these results suggest that PVT1 can promote MKL1 expression and migration of HepG2 cells through miR-3619-5p.

### PVT1 can bind to miR-3619-5p directly

**FIGURE 4:**
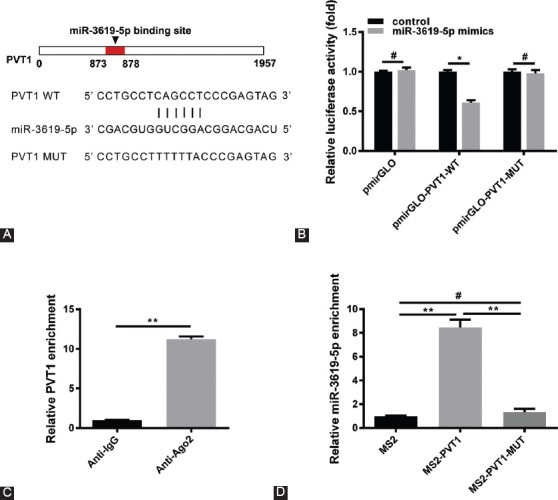
Plasma cell tumor heterotopic gene 1 (PVT1) can bind to miR-3619-5p directly. (A) Schematic representation of the predicted binding sites for miR-3619-5p in PVT1. (B) Luciferase activity in Cos-7 cells cotransfected with miR-3619-5p mimics, and luciferase reporters containing PVT1 or Mut-PVT1. Data are presented as the relative ratio of firefly luciferase activity to Renilla luciferase activity (*n* = 3, *p < 0.05, ^#^*p* > 0.05). (C) Anti-protein argonaute-2 (anti-Ago2) RNA immunoprecipitation (RIP) was performed in HepG2 cells followed by quantitative reverse transcription polymerase chain reaction to detect PVT1 association with Ago2 (*n* = 3, **p < 0.01). (D) MS2-RIP assay was used to prove the direct binding between miR-3619-5p and PVT1 in HepG2 cells (*n* = 3, **p < 0.01, ^#^*p* > 0.05).

In order to explore the interaction between PVT1 and miR-3619-5p, the sequences of PVT1 and miR-3619-5p were compared through bioinformatics (using online starBase v2.0 software, available at http://starbase.sysu.edu.cn), and the potential binding site was detected (Figure 4A). Then, two luciferase plasmids containing PVT1 (pmirGLO-PVT1-WT) and the binding site mutated form (pmirGLO-PVT1-mut) were transfected. The luciferase assay results revealed that miR-3619-5p can bind to the pmirGLO-PVT1-WT instead of pmirGLO-PVT1-mut (Figure 4B). In addition, RIP assay was performed with Ago2 antibody to determine whether PVT1 associated with the form of RNA-induced silencing complex. Data showed that PVT1 can be enriched by Ago2 antibody (Figure 4C). Furthermore, the researchers tested whether PVT1 can pull down miR-3619-5p and the MS2 RIP test was used. Subsequently, with an MS2 vector (MS2) as control, a plasmid containing the PVT1 sequence (MS2-PVT1) and its mutation type with mutated miR-3619-5p binding site (MS2-PVT1-MUT) were engineered. The qRT-PCR results showed that miR-3619-5p can be significantly enriched in the MS2-PVT1 group compared with MS2 and the MS2-PVT1-MUT group (Figure 4D). Therefore, PVT1 can bind to miR-3619-5p directly.

### MKL1 can activate the expression of PVT1 by combining with CarG box

**FIGURE 5:**
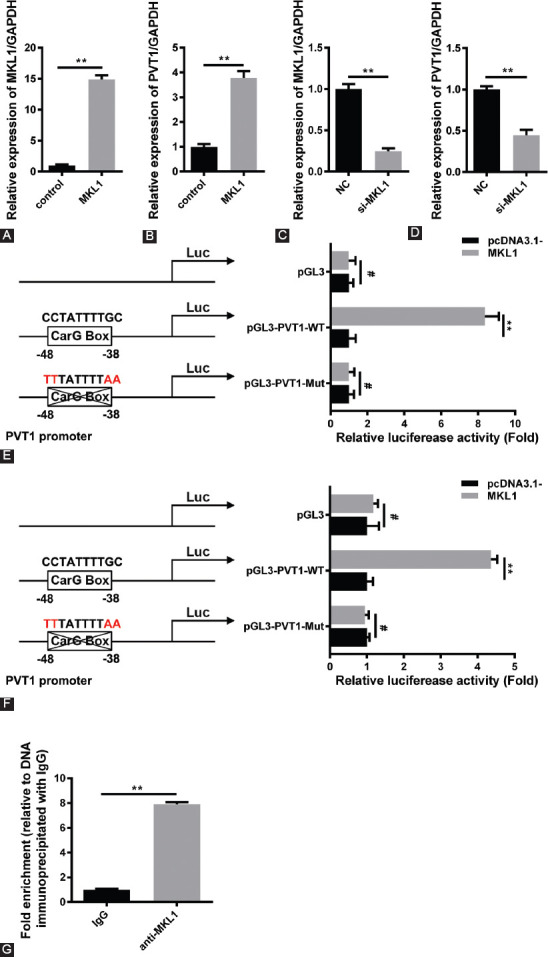
Megakaryoblastic leukemia 1 (MKL1) can activate the expression of plasma cell tumor heterotopic gene 1 (PVT1) by combining with CArG box. (A–D) After the overexpression or silencing of MKL1 in HepG2 cells, the changes in PVT1 expression were detected by quantitative reverse transcription polymerase chain reaction (*n* = 3, **p < 0.01). (E) Cos-7 cells were co-transfected with MKL1 and pGL3-PVT1-WT or mutant pGL3-PVT1-Mut plasmids, and then luciferase assay was performed. The pcDNA3.1- plasmid was used as control (*n* = 3, **p < 0.01, ^#^*p* > 0.05). (F) Luciferase assay was used to prove the binding sites between MKL1 and the promoter region of *PVT1* in HepG2 cells (*n* = 3, **p < 0.01, ^#^*p* > 0.05). (G) ChIP assay was used to determine the sites on the *PVT1* promoter where the MKL1/serum response factor (SRF) complex binds in HepG2 cells (*n* = 3, **p < 0.01).

As reported, MKL1 can regulate the expression of the target gene in the nucleus by binding to CarG box in its promoter region. Interestingly, the researchers found a CarG box in the promoter region of *PVT1* (the CarG box sequences: 5′-CCTATTTT GC-3′; position: −48–−38 of the *PVT1* promoter region). Subsequently, the results of qRT-PCR confirmed that MKL1 can activate the expression of *PVT1* (Figure 5A-D). Furthermore, the luciferase assay showed that MKL1 can activate the transcription activity of the *PVT1* promoter, while MKL1 cannot activate the transcription of *PVT1* with CArG box mutation (Figure 5E and F). Moreover, the ChIP assay also confirmed that MKL1 can combine with the CArG box of the *PVT1* promoter region (Figure 5G). These results indicate that MKL1 can activate the expression of *PVT1* by combining with CArG box.

## DISCUSSION

China is a high-incidence area of liver cancer, and the incidence of new cases accounts for about half of the global cases every year [17]. Hepatectomy is still the most important treatment for HCC in China. However, the overall curative effect after hepatectomy is still poor. The overall recurrence rate of 5 years after hepatectomy is over 80%, and the overall survival rate of 5 years is only 30–70% [18]. Intrahepatic and extrahepatic metastases are important factors affecting the long-term effect of hepatectomy. Therefore, an in-depth study of the new molecular mechanism of increased metastasis of HCC is of great significance for the design of more effective drugs to improve or supplement the surgical efficacy.

In this study, the researchers revealed a new pathway to regulate the migration of HCC cells driven by PVT1. Our results showed that MKL1 was significantly increased when PVT1 was overexpressed, resulting in enhancing the ability of migration and invasion of HepG2 cells. However, when MKL1 was silenced, PVT1 lost the ability to regulate HepG2 cell migration. These data may suggest that PVT1 can regulate the migration of HepG2 cells through MKL1. In addition, PVT1 regulated MKL1 expression by adsorption of miR-3619-5p.

The function of ncRNAs, such as microRNAs (miRNAs) and lncRNAs, in cancer and other diseases has received worldwide attention in recent years [19-24]. LncRNAs, which are longer than 200 nucleotides with limited protein-coding ability, play key roles in various diseases, particularly in cancers [25-29]. More attention has been paid to lncRNA because of the complex regulatory functions of lncRNA on gene expression (such as gene silencing, gene imprinting, chromatin modification, and post-transcriptional modifications) [30-33]. LncRNAs, such as HOX transcript antisense RNA (HOTAIR), growth arrest specific 5 (GAS5), urothelial cancer associated 1 (UCA1), HOXA transcript at the distal tip (HOTTIP), and X-inactive specific transcript (XIST), were found to play an important role in HCC at different levels [34-38]. LncRNA PVT1 is encoded by the human *PVT1* gene and is involved in the development of a variety of tumors, such as gallbladder cancer, colorectal cancer, gastric cancer, lung cancer, thyroid carcinoma, breast cancer, nasopharyngeal carcinoma, and pancreatic cancer [39-48]. Thus, PVT1 has the potential to become a molecular target for the clinical diagnosis and treatment of tumors. PVT1 was confirmed to be overexpressed in HCC and associated with poor prognosis, which suggested that PVT1 may play an important role in the development of HCC [49]. In this study, the researchers confirmed that PVT1 can promote the migration of HCC cells, consequently revealing a new molecular mechanism.

The function and mechanism of lncRNAs are complex. There is a lot of evidence showing that lncRNAs can cause target gene overexpression by functioning as a ceRNA sponge to miRNAs, suppressing the binding of miRNAs to the target mRNAs and regulating their function [50,51]. For example, lncRNA small nucleolar RNA host gene 20 (SNHG20) induces gastric cancer progression via upregulating zinc finger protein X-linked (ZFX) expression by absorbing miR-495-3p [52]. LncRNA activated by transforming growth factor-b (ATB) contributes to Twist1 expression and breast cancer epithelial–mesenchymal transition (EMT) through inhibiting miR-200c [53]. For C5orf66-AS1, miR-637 is a target miRNA in the process of cervical cancer growth [54]. In this study, the FISH results have indicated that PVT1 was partly located in the cytoplasm of HepG2 cells, suggesting that PVT1 may work as a ceRNA for some miRNAs. Through bioinformatics analysis, the researchers identified a potential target miR-3619-5p, which may be a regulator of MKL1. Finally, we showed that PVT1 can sponge miR-3619-5p from MKL1 mRNA to enhance metastasis of HCC cells.

**FIGURE 6:**
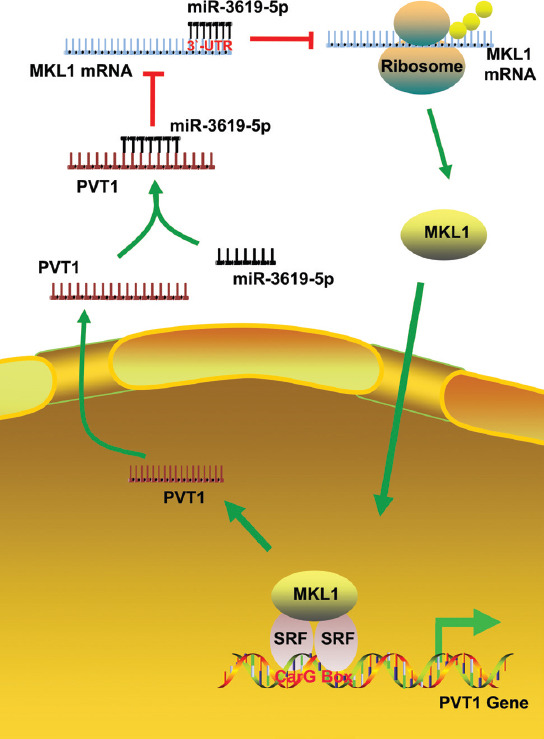
Schematic representation of the effect and molecular mechanism of plasma cell tumor heterotopic gene 1 (PVT1) in the process of regulating the migration of hepatocellular carcinoma (HCC) cells. PVT1 can sponge miR-3619-5p from megakaryoblastic leukemia 1 (MKL1) mRNA to enhance the metastasis of HCC cells. In addition, MKL1 can reverse-activate the expression of PVT1 by forming a complex with serum response factor (SRF) and by binding to the CArG box in the promoter region of *PVT1*.

Collectively, our data revealed a new kind of regulation mechanism led by PVT1. PVT1 can upregulate the expression of MKL1 via competitive sponging miR-3619-5p to exhibit oncogenic activity, which may be a novel signaling pathway for HCC treatment. Finally, the study confirmed that MKL1 can reverse-activate the transcription of PVT1 (Figure 6) and MKL1 and PVT1 formed a positive feedback loop that contributed to tumor migration.
